# An Integrated Health Monitoring Method for Structural Fatigue Life Evaluation Using Limited Sensor Data

**DOI:** 10.3390/ma9110894

**Published:** 2016-11-04

**Authors:** Jingjing He, Yibin Zhou, Xuefei Guan, Wei Zhang, Yanrong Wang, Weifang Zhang

**Affiliations:** 1 School of Reliability and System Engineering, Beihang University, Beijing 100191, China; hejingjing@buaa.edu.cn (J.H.); zhangweifang@buaa.edu.cn (W.Z.); 2 School of Energy and Power Engineering, Beihang University, Beijing 100191, China; zhouyibin@buaa.edu.cn (Y.Z.); yrwang@buaa.edu.cn (Y.W.); 3 Siemens Corporation, Corporate Technology, 755 College Rd. E., Princeton, NJ 08540, USA; xf.guan@gmail.com

**Keywords:** limited sensor data, structural health monitoring, empirical mode decomposition, fatigue life evaluation

## Abstract

A general framework for structural fatigue life evaluation under fatigue cyclic loading using limited sensor data is proposed in this paper. First, limited sensor data are measured from various sensors which are preset on the complex structure. Then the strain data at remote spots are used to obtain the strain responses at critical spots by the strain/stress reconstruction method based on empirical mode decomposition (REMD method). All the computations in this paper are directly performed in the time domain. After the local stress responses at critical spots are determined, fatigue life evaluation can be performed for structural health management and risk assessment. Fatigue life evaluation using the reconstructed stresses from remote strain gauge measurement data is also demonstrated with detailed error analysis. Following this, the proposed methodology is demonstrated using a three-dimensional frame structure and a simplified airfoil structure. Finally, several conclusions and future work are drawn based on the proposed study.

## 1. Introduction

Structural health monitoring (SHM) is an effective tool to monitor the status of a target system for maintenance purposes [1,2]. Moreover, SHM has drawn a lot of attention in the SHM community due to flexibilities of smart sensors and advances in composite materials [3,4,5,6]. For most mechanical systems subjected to fatigue loads, fatigue crack propagation is one of the major failure mechanisms, and fatigue damage is mainly dependent on the applied stress history under realistic conditions. The practical challenge for fatigue life prediction in such cases is that the stress and fatigue load in the vicinity of the fatigue crack is very difficult to measure. Therefore, one of the critical aspects in fatigue life prediction analysis is the stress information. The knowledge of the stress information of a structure allows for reliable and accurate fatigue life prediction. It is frequently seen that the critical spots for fatigue damage usually have no direct sensor measurements. Using measurements from pre-installed sensors to infer the information about the critical spot—i.e., a fatigue crack-damaged location—without direct sensor measurements may be the only practical option in realistic situations. Therefore, extrapolation to a critical spot from measurements located in a remote spot is a mandatory step for fatigue prognostics and the remaining life prediction.

Recent advances of dynamical response reconstruction methods include frequency-domain methods and direct time-domain methods [7,8,9]. However, very few published works have been found for fatigue life evaluation using limited sensor data. Direct time-domain reconstruction of dynamical responses for acceleration and velocity using the empirical mode decomposition (EMD) method is demonstrated in [10]. For convenience, the reconstruction methods based on the EMD and the finite element modeling (FEM) of the structure is abbreviated to REMD in this paper. One of the objectives of the proposed reconstruction methodology is to provide accurate stress information for fatigue life prediction using sparse and remote strain gauges installed in the structure. The proposed reconstruction methodology can be readily integrated into existing structural health monitoring systems for prognostics.

Stress responses of a location of interest can be obtained whereby the strain responses for the location are reconstructed using the REMD method with remote strain gauge measurements. The reconstructed stress responses form a spectrum loading for the location of interest. In practice, the spectrum loading for a mechanical part is a complex and often stochastic sequence of load amplitudes. The calculations of fatigue life are different between crack-damaged and normal mechanical parts. For a component without fatigue cracks, the calculation of fatigue life based on the S-N curve [11] and Miner’s rule [12] can be adopted. In such cases, the following procedure is widely used:

1. Reduce the stochastic loading spectrum to a set of simple cyclic loadings using the rainflow counting method [13,14].
2. Perform statistical analysis for the results from the rainflow analysis to create a histogram of cyclic stress and form a fatigue damage spectrum.
3. For each stress level in the fatigue damage spectrum, calculate the degree of cumulative damage using the S-N curve (of the material).
4. Combine the damage contributions of each stress level using Miner’s rule.

Additionally, a number of interesting studies of fatigue life evaluation based on strain field measurements have been raised in recent years. A fast and reliable algorithm for automatic simulations of crack propagation in bi-dimensional and non-planar shell FE models is described in [15]. Giglio [16] performed some damage-tolerant behavior verification tests on full-scale panel Al-lithium alloy specimens instrumented with several strain gauges to obtain a strain map. He monitored the propagation of the crack caused by artificial damage until the progressive failure of the panel reached one or more stringers. Luo [17] investigated an energy-based multiscale damage criterion for a biaxial loading case and conducted numerical fatigue analysis which showed that the multiscale fatigue damage model can provide acceptable prediction of failure. A damage tolerance analysis is also implemented using an FEM-based approach combined with a 3D automatic crack propagation algorithm in [18]. Sbarufatti [19] applied sequential Monte-Carlo sampling to estimate the probabilistic residual life of a structural component subjected to fatigue crack propagation, while real-time estimation of crack length is provided through a committee of artificial neural networks, trained with finite element-simulated strain patterns. However, most of these approaches require cycle-counting techniques to transform the direct stress history to cycle history before the fatigue analysis can be performed. In engineering, loading history is usually complex or even far from being cyclic; generally, the conversion from random load history to the cycle load sequence significantly modifies the load history. Therefore, an integrated health monitoring approach for structural fatigue life evaluation with real-time spectrum loading is proposed in this paper. Fatigue crack growth models reported in [20,21,22] which changed the cycle-counting to small time counting can deal with stochastic spectrum loadings and the reconstructed stress responses can be directly used.

The objective of the study is to develop a novel method for fatigue life evaluation using limited measurements from usage monitoring system. The proposed method extends the previous EMD-based time-domain reconstruction method by using the finite element model to derive a strain transformation function in modal coordinates. EMD method with intermittency criteria is employed in the proposed method to decouple the remote strain measurements into modal coordinates, and the extrapolation of the strain and stress responses is made using the strain and stress transformation function. Mode superposition is used to obtain the strain and stress responses of the critical spot for fatigue life prediction. One potential benefit is that the fatigue damage can be obtained concurrently with structural dynamics analysis, which is critical for real-time decision making.

**Figure 1:**
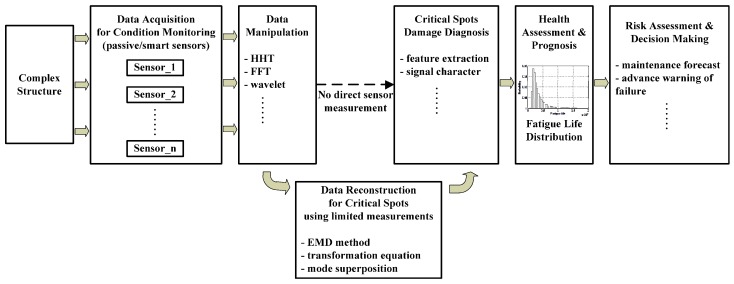
Overall procedure of the fatigue life evaluation method.

Figure 1 presents the overall fatigue life evaluation method. First, limited sensor data are measured from various sensors which are preset on the complex structure. Then the strain data at remote spots are used to obtain the strain responses at critical spots. All the computations in this paper are directly performed in the time domain. After the local stress responses at critical spots are determined, fatigue life evaluation can be performed for structural health management and risk assessment.

The paper is organized as follows. First, the EMD method for signal decomposition is briefly introduced, and the required signal filtering process using the intermittency criteria is discussed. Next, the transformation equation for strain and stress is derived using the FE model of the structure. Following this, the fatigue life prediction using the reconstructed stress is calculated and compared with theoretical solutions. A three-dimensional frame structure and a simplified airfoil structure are used to demonstrate and validate the proposed method. Finally, conclusions are drawn based on the current study.

## 2. Integrated Health Monitoring Method Development

The proposed integrated health monitoring methodology is demonstrated in this section. The overall process for the reconstruction involves several steps, as illustrated in [10]. Details of each of the calculation steps are discussed in this section.

### 2.1. Strain/Stress Reconstruction Method Based on Empirical Mode Decomposition

An essential step of the proposed dynamic response reconstruction method is to obtain the individual modal responses of the measured signals. The EMD method with intermittency criteria is adopted to decompose the measured signals into modal responses which are called intrinsic mode functions (IMFs) in EMD. In this section, the standard sifting process of the EMD method is briefly discussed.

Using those modal responses, the modal responses at inaccessible locations are computed with the mode shapes. Then modal superposition methodology is employed to calculate the dynamic responses in time domain after all the modal responses have been obtained. To ensure that each IMF contains only one frequency component, the sifting process with intermittency criteria is used. This method is very efficient in terms of computational cost, and very suitable for various dynamic response reconstructions based on the different types of sensor measurements. A limited number of measurement locations are required.

By using the EMD method with intermittency criteria discussed in [10] for different natural frequencies, all modal responses can be obtained. These IMFs have several characteristics: (1) Each IMF contains the intrinsic characteristics of the signal; (2) Once an IMF is obtained, the next IMF will not have the same frequency at the same time instant [23,24]; and (3) The first IMF for each IMF series is considered the approximation of modal response. Using the sifting process with intermittency criteria, the original signal expression can be written as Equation (1).

(1) $$y(t)\approx \sum_{i=1}^{m}x_{i}(t)+\sum_{i=1}^{n-m}f_{i}(t)+r(t)$$

where the original signal y(t) can be expressed as the summation of *n* IMFs and a residue term r(t) by applying the standard EMD method. $x_{i}(t)$ is the *m* modal response (that is also an IMF) for the *i*th mode. Terms $f_{i}(t)$(i=1,⋯n−m) are other IMFs but not modal responses.

For a general structure, finite element model (FEM) can be used as the structural model to derive transformation equations. Considering a general FEM to describe the structure under analysis, the system dynamics equation can be expressed as

(2) $$M\ddot{X}+C\dot{X}+KX=F$$

where **M**, **K** and **C** are mass, stiffness, and damping matrices, respectively. **X** is the displacement vector and **F** is the load vector. For practical structures subject to stochastic excitations, **F** is unknown and directly solving Equation (2) to obtain the dynamical responses of a sensor from an inaccessible location is not possible. However, the mode superposition method allows for correlating displacement responses of two different degree of freedoms (DOFs) in the modal coordinates through the mode shape matrix. The mode shape matrix can readily be obtained by solving the eigenvalue problem of

(3) $$[\Phi ,\lambda ]=\mathrm{eig}([M^{-1}K])F$$

where **Φ** and ***λ*** are the eigenvectors and eigenvalues, respectively. **Φ** (Equation (4)) is also referred to as the mode shape matrix. ***λ*** corresponds to the natural frequencies of the structure, i.e., $\lambda ={\left(2\pi f\right)}^{2}$ where **f** is the vector of natural frequencies.

(4) $$\Phi =\left[\begin{array}{ccc} \varphi_{11} & \cdots & \varphi_{n1}\\ \vdots & \ddots & \vdots \\ \varphi_{1n} & \cdots & \varphi_{nn} \end{array}\right]$$

The physical meaning of **Φ** can be interpreted as follows: each column of **Φ** represents a mode and each component in the column represents the displacement contribution of a DOF in the structure.

The transformation equation for strain responses under *i*th mode between two elements indexed by *e* and *u* can be obtained by using Equation (6) as

(5) $$\frac{B^{\left(e\right)}\Phi_{i}^{\left(e\right)}}{B^{\left(u\right)}\Phi_{i}^{\left(u\right)}}=\frac{\alpha B^{\left(e\right)}\delta_{i}^{\left(e\right)}}{\alpha B^{\left(u\right)}\delta_{i}^{\left(u\right)}}=\frac{\eta_{i}^{\left(e\right)}}{\eta_{i}^{\left(u\right)}}$$

where $B^{\left(k\right)}$ is the strain-displacement matrix for element *k* and $\Phi_{i}$ is the *i*th column vector in the mode shape matrix Φ, $\eta_{i}^{\left(k\right)}$ is the strain response vector associated with *i*th mode and *k*th element under modal coordinates, α is a scalar constant for a given time index *t*. The result of Equation (5) indicates that if the physical strain responses are measured at one location (represented by element *e* in the FE model), it can be decomposed to its modal responses, i.e., $\varepsilon^{\left(e\right)}\approx \sum_{i=1...m}\eta_{i}^{\left(e\right)}$, the physical strain responses at a sensor inaccessible location (represented by element *u* in the FE model), can be reconstructed using the following transformation equation:

(6) $$\varepsilon^{\left(u\right)}(t)\approx \sum_{i=1...m}\left[\eta_{i}^{\left(e\right)}(t){\left(\frac{B^{\left(e\right)}\Phi_{i}^{\left(e\right)}}{B^{\left(u\right)}\Phi_{i}^{\left(u\right)}}\right)}^{-1}\right]$$

where i=1...m denotes the participating modes and all other notations are defined as before. Once the physical strain responses are reconstructed using Equation (6), the stress responses can be readily calculated using the following constitutive equation:

(7) σ(t)=cε(t)

where **c** is the material matrix.

Once the strain and stress responses of locations of interest are obtained using the proposed reconstruction method, the stress responses can be used in the fatigue crack growth models based on fracture mechanics or the concept of S-N curves. The fatigue life prediction integrating the strain and stress reconstruction method is presented next.

### 2.2. Fatigue Life Prediction Integrating Strain and Stress Reconstruction Method

The fatigue crack growth model used in this study is a small time scale model. Since the development of the fatigue crack growth model is beyond the scope of this paper, only a brief introduction is given here. Detailed derivation and model validation for the new material fatigue crack growth model can be found in the referred article [25].

**Figure 2:**
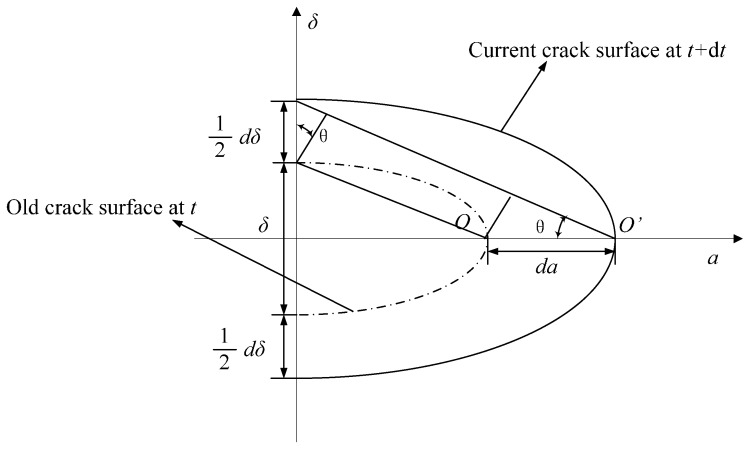
Schematic representation of crack tip geometry profiles during fatigue crack growth.

The small time scale model is developed based on the geometric relationship between the crack tip opening displacement (CTOD) and the instantaneous crack growth kinetics. The geometric relationship between the CTOD and the instantaneous crack growth kinetics is shown in Figure 2. The schematic illustration in Figure 2 is for a through thickness crack in an infinite plate. Only the tip region is shown. As shown in Figure 2, the crack will extend a distance (*da*), after a small time increment (*dt*) and the crack tip will extend from O to O’. Considering the geometry of crack tips at two time points (*t* and *t* + *dt*), the crack growth rate *da*/*dt* for an infinitesimal crack growth is derived as it is shown in Equation (8), where *θ* is the crack tip opening angle (CTOA).

(8) $$da=\frac{\cot(\theta )}{2}d\delta =Cd\delta$$

The CTOD can be approximately expressed as Equations (9) and (10) using the plastic zone model proposed by Irwin [26]. Equation (9) is for elastic-perfect-plastic material behavior and ignores the hardening effect [27].

(9) $$\delta =\frac{1}{2}\frac{K^{2}}{E\sigma_{y}}=\lambda \sigma^{2}a$$

(10) $$\lambda =\frac{\pi }{2E\sigma_{y}}$$

where *E* is the Young’s modulus. *σ_y_* is the yield strength. It should be noted that *σ* in Equation (9) is the nominal stress. According to Figure 2, the crack is propagated from O to O’ during a small time increment. The crack length increment is *da* and the CTOD increment is *dδ*. The CTOD increment *dδ*, is expressed as Equation (11)

(11) $$d\delta =\lambda (2\sigma ad\sigma +\sigma^{2}da)$$

Substituting Equation (11) into Equation (8) and dividing on both sides by a small time increment *dt*, the instantaneous crack growth rate is represented as

(12) $$\frac{1}{C\lambda a}\frac{da}{dt}=\frac{2\sigma }{1-C\lambda \sigma^{2}}\frac{d\sigma }{dt}$$

The proposed methodology describes crack growth rate in terms of time scale instead of cycle, according to Equation (12). The crack length at any arbitrary time can be calculated by direct time integration. The above discussion is for the case when the crack starts to grow. The crack may not grow during the entire duration of the cyclic loading. A general expression considering the non-uniform crack growth is expressed as

(13) $$\dot{a}=H\left(\dot{\sigma }\right)\cdot H\left(\sigma -\sigma_{ref}\right)\cdot \frac{2C\lambda }{1-C\lambda \sigma^{2}}\cdot \dot{\sigma }\cdot \sigma \cdot a$$

where “ ˙ ” denotes time derivative throughout this paper. *H* is the Heaviside function. *σ_ref_* is the reference stress level where the crack starts to grow. Cracks do not grow during the entire duration of the cyclic loading. For example, crack does not grow during the unloading path due to the energy principle. Also, crack only starts to grow when the applied loading is beyond a certain stress level. The crack length needs to be calculated by performing the integration from the lower integration limit (e.g., the time when the reference stress level is reached) to the upper integration limit (e.g., the time when the maximum stress level is reached). The crack closure model is essentially contained in the calculation of reference stress level. In this case, the determination of the reference stress level is critical in order to perform the integration. The small time scale model has been validated using the existing experimental data for various materials under both constant loading and variable amplitude loadings [25]. There are several advantages for the developed small time scale model: (1) Comparing with Paris’s model, the small time scale model does not need cycle-counting (e.g., only using direct time domain integral) under random variable loadings. Paris’s model considers the average crack growth per cycle and cannot include the detailed mechanisms within one loading cycle (e.g., non-uniform crack growth kinetics within one cycle as shown in Equation (13); (2) Stress ratio effect has been included in the small time scale model since the direct stress state instead of stress range is used; and (3) One unique advantage of the small time scale model is that it can be seamlessly coupled with structural analysis and has great potential for concurrent structural dynamic analysis and multilevel (e.g., structural level and material level) fatigue life evaluation. This capability makes it an ideal model for real-time damage analysis and on-line decision-making [21].

## 3. Example Validation

Two numerical examples are presented here. The first example is a three-dimensional frame structure subject to random forces. The second example is a complex airfoil structure with 19,956 DOFs. These two examples demonstrate the reconstruction methodology integrating the fatigue damage life evaluation.

### 3.1. A Three-Dimensional Frame Structure Example

**Figure 3:**
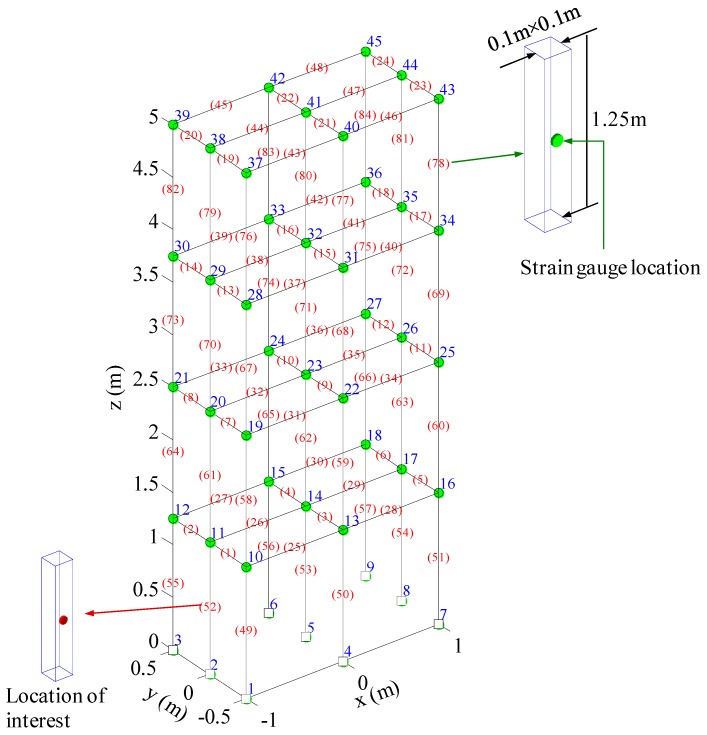
FE model of the spatial frame structure.

The proposed methodology can be directly applied to structural level reconstruction analysis. To demonstrate the basic idea, a 216-DOF spatial frame structure is used here. The finite element model diagram of the structure and its dimensions are shown in Figure 3. The structure is a four-story steel structure and it has extents of 2 m, 1 m, and 5 m in the x-axis direction, y-axis direction, and z-axis direction, respectively. Each story has a height of 1.25 m. The figure nodes are labeled using nearby numbers and elements are labeled with numbers in round brackets. Each of the elements has two nodes and 12 DOFs in total. Each node has six DOFs, namely the displacements of x, y, and z directions and the rotations along x, y, and z axes. All the nodes attached to the ground (i.e., z = 0) are prescribed and the structure has 216 DOFs. Required properties of the structural member element for FE modeling are listed in Table 1.

At each floor, stochastic forces in the x-axis direction are applied to simulate the ambient excitations. The surface geometry center of the element (78) is used as the location of the actual strain gauge. Sensor measurements are obtained by solving the dynamical equations of the finite element model and three different (2%, 5%, 10%) RMS noise terms are added to the deterministic results to represent the measurement uncertainty. The sampling frequency is 1000 Hz. To simulate the realistic measured signals, the noisy components are of Gaussian pulse processes with root mean square (RMS) setting to a percentage of the largest RMS of the measured signal. Then the noise components can be quantified by the noise level in this paper. For example, a 5% noise level is to generate noise components from Gaussian pulse processes with RMS setting to 5% of the largest RMS of the strain responses. Without loss of generality, the element (52) is arbitrarily chosen to represent the location of interest. For this element, strain and stress responses at the geometry center of the surface perpendicular to the y-axis direction can have maximum response amplitudes and this geometry center is the location of interest for reconstruction. Both the strain gauge measurement location and the reconstructed location are shown in Figure 3. For illustration purposes, only the normal strain and stress are considered to be significant. The reconstruction for shear strain and stress responses can be obtained using the same methodology.

**Figure 4:**
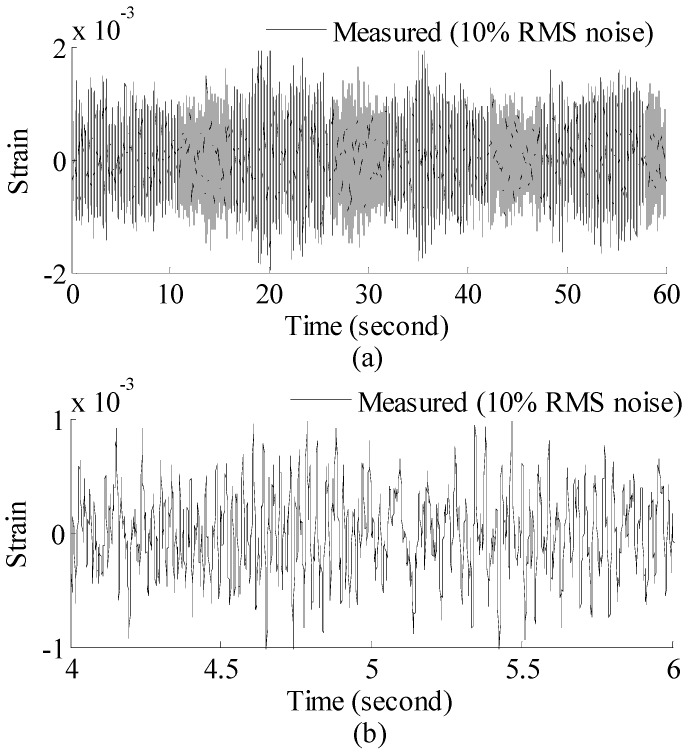

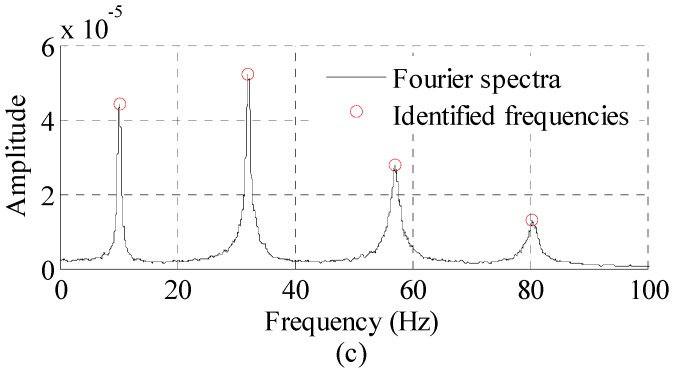
Strain gauge measurement data and Fourier spectra of the data. (**a**) Strain gauge measurement data (0–60 s); and (**b**) concentrated on 4–6 s for clear presentation; and (**c**) Fourier spectra of the measurement data (0–60 s).

**Figure 5:**
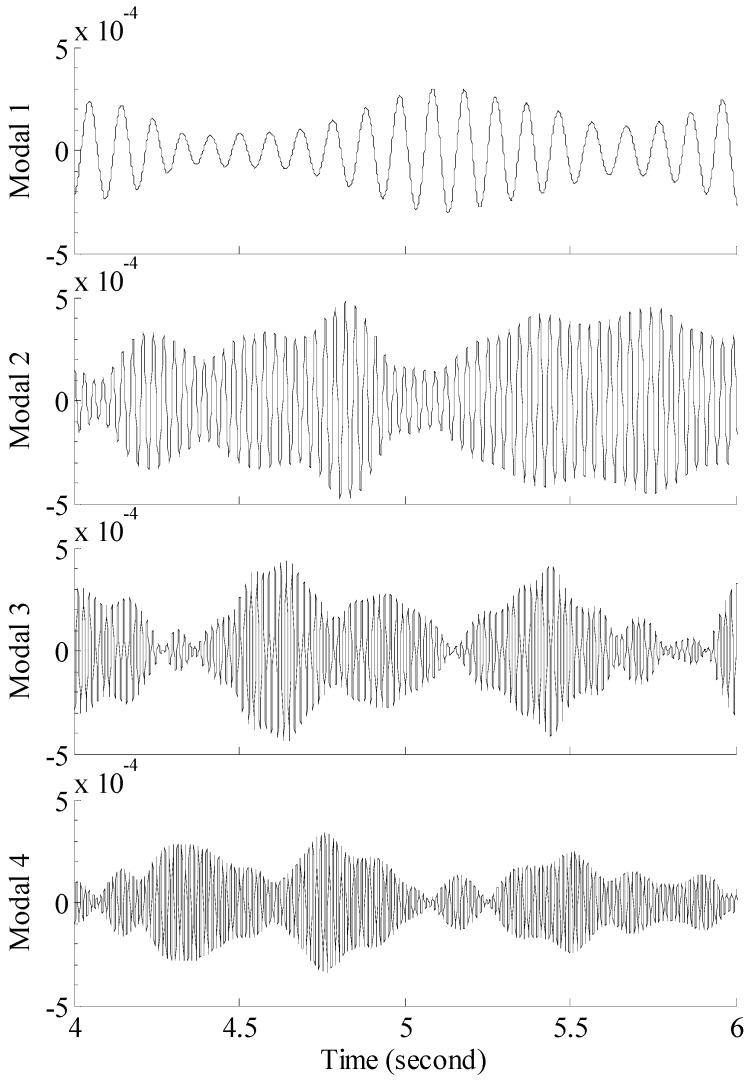
Modal responses of the strain gauge measurement data obtained by EMD method with intermittency criteria. **Model 1:** modal response for the 1st mode; **Model 2:** modal response for the 2nd mode; **Model 3:** modal response for the 3rd mode; **Model 4:** modal response for the 4th mode.

**Figure 6:**
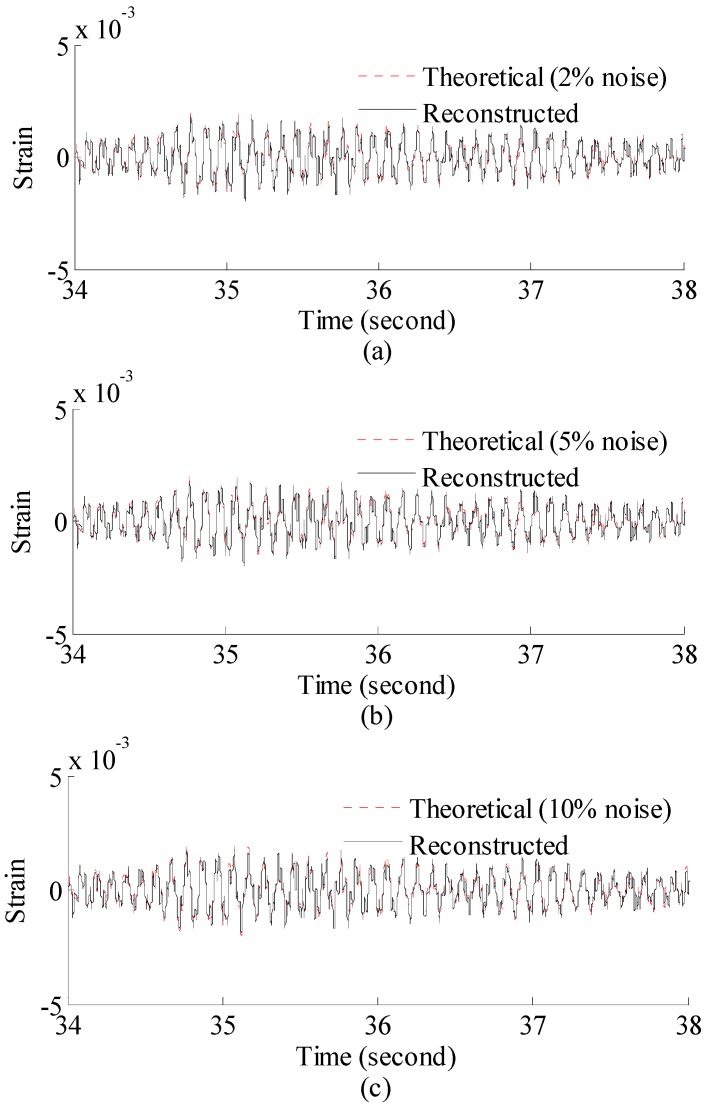
Reconstructed and theoretical strain responses for the location of interest under (**a**) 2%; (**b**) 5%; (**c**) 10% noise level. Results are concentrated on 34–38 s for clear presentation.

**Figure 7:**
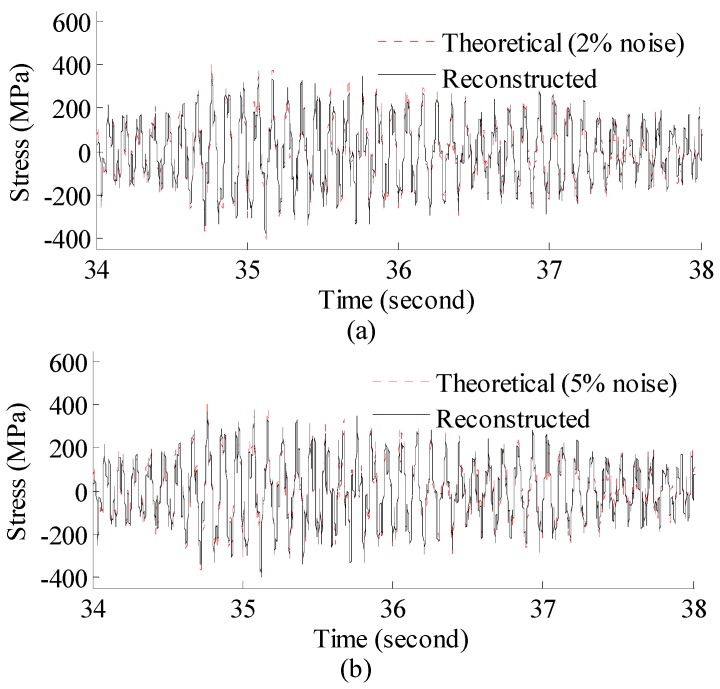

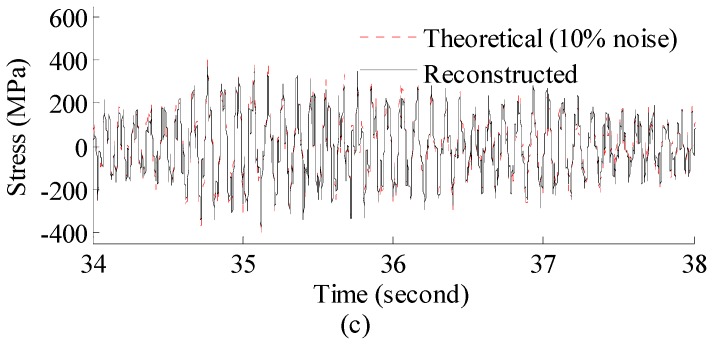
Reconstructed and theoretical bending stress responses for the location of interest under (**a**) 2%; (**b**) 5%; (**c**) 10% noise level. Results are concentrated on 34–38 s for clear presentation.

The 60 s of synthesized strain gauge measurement data with 5% RMS noise terms are presented in Figure 4a. The Fourier spectra of the strain gauge measurement data indicate four significant frequencies as shown in Figure 4c. Those four frequencies are used to design band-pass filters for the modal responses extraction. Figure 5 presents the modal responses obtained by the EMD method with intermittency criteria. The reconstructed strain gauge responses for the location of interest in element (52) and the theoretical results under 2%, 5%, 10% noise level are compared in Figure 6. The reconstructed stress responses based on the reconstructed strain results under three noise levels are shown in Figure 7. The correlation between the reconstructed strain responses and the theoretical strain calculation results is 0.9789, 0.9788, 0.9785. The reconstructed responses under the three noise levels suggests that the reconstructed responses are very consistent, which indicates that the presented integrated health monitoring method is robust against noisy measurement signals.

### 3.2. A Simplified Airfoil Structure Model Example

**Figure 8:**
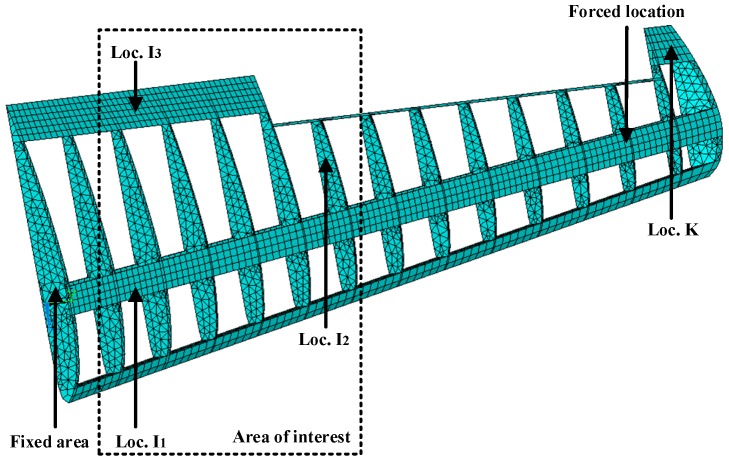
FE model of the simplified airfoil structure.

The proposed methodology can be directly applied to structural level reconstruction analysis. To demonstrate the basic idea, a 19956-DOF simplified airfoil structure model is used here. The finite element model diagram of the structure and its dimensions are shown in Figure 8. The structure is a simplified airfoil without skin and it has extents of 3.49 m, 0.11 m, and 0.95 m in the x-axis direction, y-axis direction, and z-axis direction, respectively. The element type is solid185 so that it is automatically meshed with hexahedron or (reduced) tetrahedron elements in ANSYS. Each of the elements has eight nodes and 24 DOFs in total. Each node has three DOFs, namely the displacements of x, y, and z directions. All the nodes attached to the airframe (i.e., z = 0) are prescribed and the structure has 19956 DOFs. Required properties of the FE modeling in ANSYS are listed in Table 2.

Near the end of the airfoil (node 3224 in FE model), transient force with the amplitude of 1000 N in the y-axis direction is applied to simulate the ambient excitations for the first 0.01 s. The location K (element 1847 in FE model) in Figure 8 is used as the location of the actual strain gauge. Sensor measurements are presented by transient dynamic analysis in ANSYS and 5% RMS noise terms are added to the deterministic results to represent the measurement uncertainty. The sampling frequency is 1000 Hz. For the airfoil example, the fixed end of the airfoil (Loc. I_1_, I_2_, I_3_) are chosen as points of interest for reconstruction. The strain gauges are placed near the free end of the airfoil (Loc. K) where the sensors are easy to set. The proposed method provides a fundamental tool to reconstruct the stress information of critical spots using remote and sparse sensor measurements. The location of critical spots can be inferred based on engineering experience and numerical simulations. Without loss of generality, the locations I_1_, I_2_, I_3_ (element 1082, 2558, 3043 in FE model) in Figure 8 are arbitrarily chosen to represent the location of interest for reconstruction. Both the strain gauge measurement location and the reconstructed location are shown in Figure 8.

#### 3.2.1. Strain and Stress Response Reconstruction

**Figure 9:**
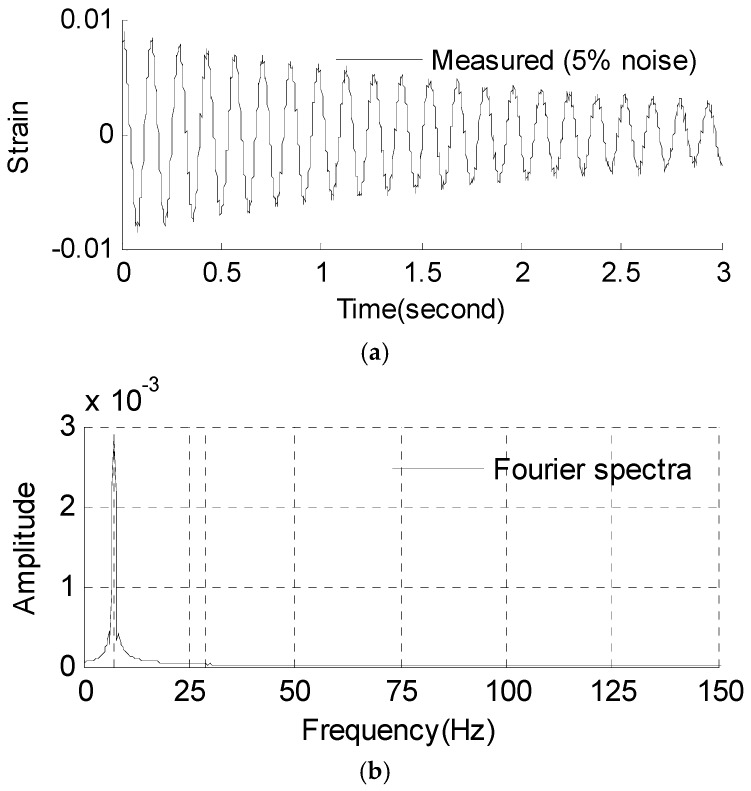
Strain gauge measurement data and Fourier spectra of the data. (**a**) Strain gauge measurement data (0–3 s); and (**b**) Fourier spectra of the measurement data (0–3 s).

**Figure 10:**
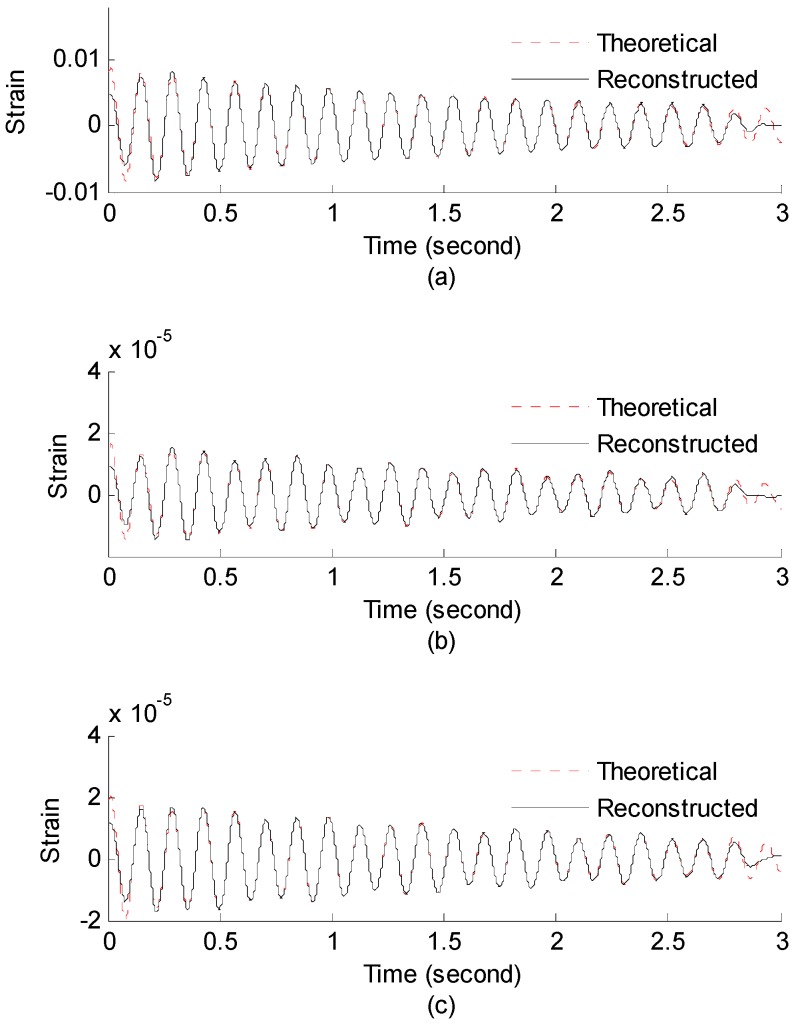
Reconstructed and theoretical strain responses for (**a**) the location I_1_; (**b**) the location I_2_; (**c**) the location I_3_.

**Figure 11:**
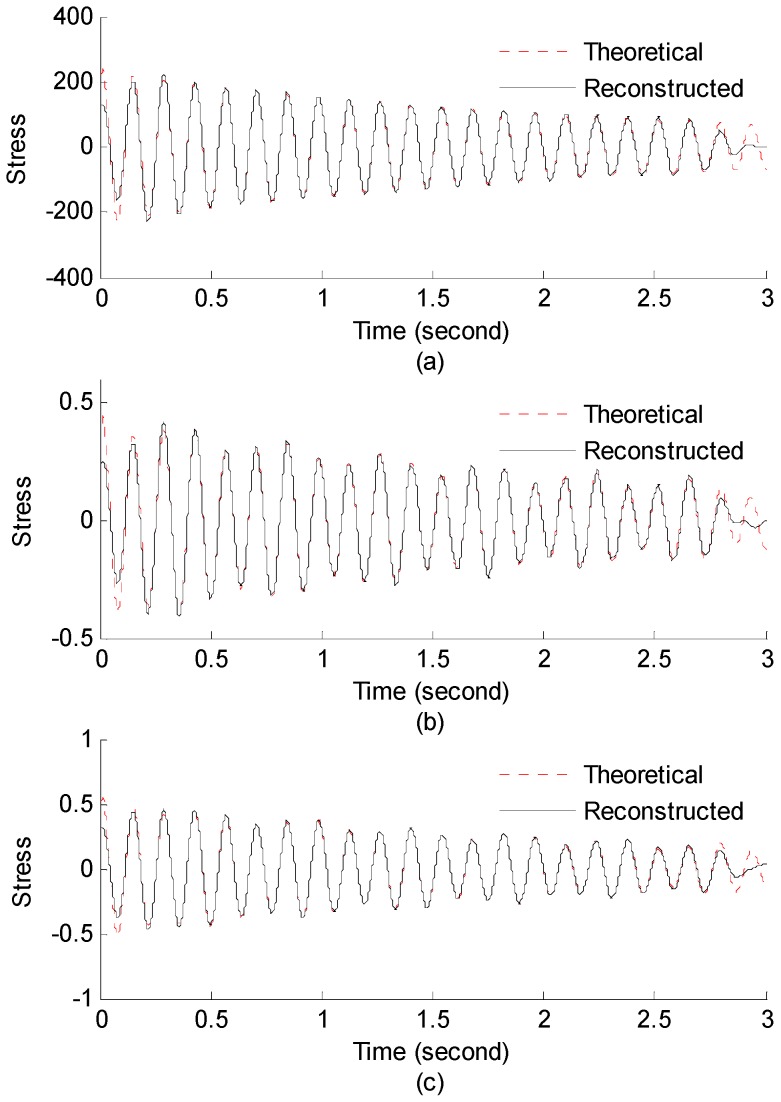
Reconstructed and theoretical bending stress responses for (**a**) the location I_1_; (**b**) the location I_2_; (**c**) the location I_3_.

Three seconds of synthesized strain gauge measurement data with 5% RMS noise terms are presented in Figure 9a. The Fourier spectra of the strain gauge measurement data indicate a significant frequency component as shown in Figure 9b. The frequency is used to design the band-pass filter for the modal responses extraction. The reconstructed strain gauge responses for the locations of interest I_1_, I_2_, I_3_, and the theoretical results are compared in Figure 10. The reconstructed stress responses based on the reconstructed strain results are shown in Figure 11. The correlation between the reconstructed strain responses and the theoretical strain calculation results is 0.9849, 0.9867, and 0.9865 respectively.

The discrepancies on the right end side of Figure 10 and Figure 11 are caused by the Gibbs phenomenon which has also been referred to as boundary/end effect in the EMD method [28], which has been widely discussed as the intrinsic weakness of EMD. The solution and verification of the Gibbs phenomenon is also worthy of future research. This is also a limitation of the method. Some clarification is discussed below in the conclusions.

**Figure 12:**
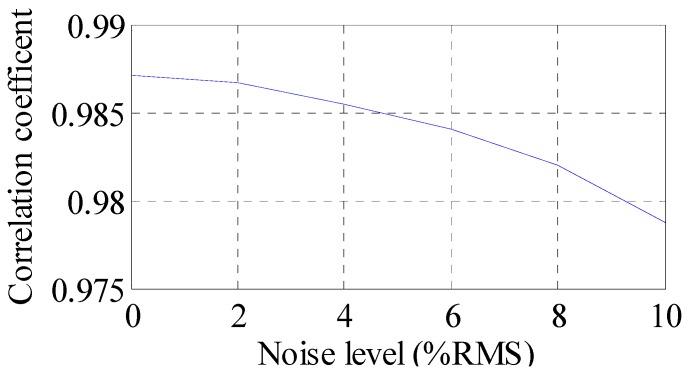
Reconstruction performance measured in correlation coefficient under different noise levels.

To investigate the effect of measurement noise level to the reconstructed results, several numerical cases with various noise levels are studied. The noise components are added to the strain responses and the results are used as the representative noisy strain measurement data. Correlation coefficient is used as a metric to evaluate the similarity between the theoretical responses and the reconstructed responses for bending stresses. RMS is set to taking value from 0% to 10% with 1% increment. At each of the RMS settings, bending stress responses at location I_1_ (as shown in Figure 8) are reconstructed based on the strain measurement at location K (as shown in Figure 8). The correlation coefficient between the reconstructed stress responses at location I_1_ and the theoretical stress responses (also with noise components) at location I_1_ is calculated. Results for all RMS settings are presented in Figure 12.

It can be seen that the performance is degraded with the increase of noise level. The overall performance of the reconstruction method is larger than 97.5% when noise levels are not larger than 10% RMS in this example. Next, fatigue life evaluation integrating the reconstructed stress responses is presented.

#### 3.2.2. Fatigue Life Evaluation Using the Reconstructed Stress Responses

**Figure 13:**
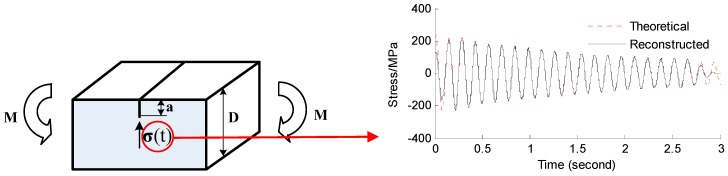
Single edge notch bending solid segment.

To demonstrate the fatigue life evaluation with reconstructed stress responses, a numerical crack growth analysis using the original and reconstructed signal with different noise levels in the airfoil structure model example are performed. The strain gauges are placed at the location K, while the location I_1_ is the point of interest. A mode I planar crack is considered in the demonstrated examples which is reduced to a single edge notch bending problem as shown in Figure 13.

**Figure 14:**
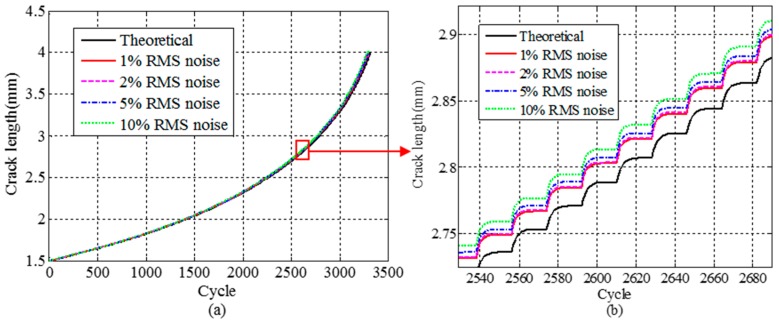
Crack growth trajectories calculated using theoretical and reconstructed stress responses under different RMS noise levels. (**a**) Entire 3500 cycles crack growth trajectories; (**b**) crack growth trajectories (2532–2684 cycles).

**Figure 15:**
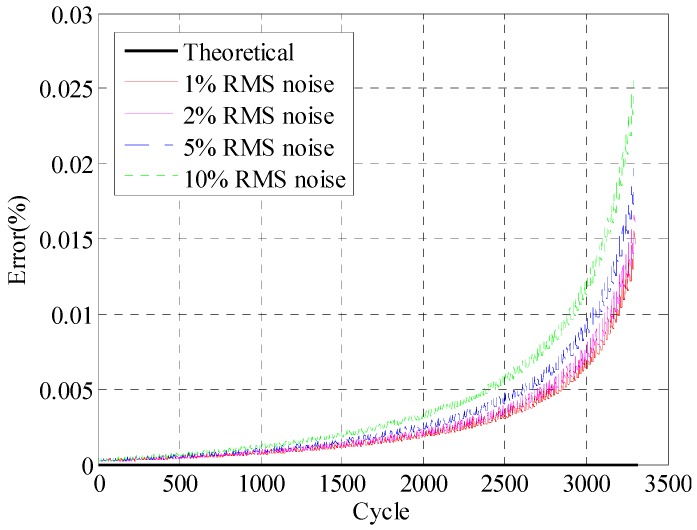
Error analysis of crack length growth evaluation using theoretical and reconstructed stress responses under different RMS noise levels.

The initial crack is assumed to be 1.5 mm at location I_1_ in Figure 13. The given periodic force which is composed of normal stress in Figure 11 is applied at location I. For clear demonstration, the crack size versus number of cycles (a-N) curves are used for fatigue crack growth trajectory demonstration in Figure 14. Figure 14a presents the overall fatigue crack growth evaluation results using both theoretical and proposed REMD methods. Four different noise levels (1%, 2%, 5%, 10% RMS noise) are used to verify the robustness of the proposed method. Results of 2532 to 2684 cycles are also shown in Figure 14b for clear presentation of fatigue crack growth behavior. The x-axis represents the cycles in the numerical simulation. The y-axis is the simulated crack length using the proposed small time scale fatigue crack growth model. As shown in Figure 14, all crack length curves agree well with each other. In the current study, the noisy components are Gaussian pulse process with root mean square (RMS) setting to a percentage of the largest RMS of the measured signal. Therefore, as the percentage of the noise increases, it may cause a larger peak of the strain response, which will lead to an increase of the crack growth rate. Quantitative error analysis is performed and shown in Figure 15. The x-axis represents the time points. The y-axis is the maximum absolute error of the crack length prediction normalized by the crack length. The error definition is

(14) $$Error=\frac{\max|\hat{a}(t)-a(t)|}{a_{e}(t)}$$

where $\hat{a}(t)$ is the crack length at time t using reconstructed stress responses. a(t) is the crack length at time t using theoretical stress responses. $a_{e}(t)$ is the final crack length of calculation. It is shown that the prediction error of crack length does not have a monotonic trend with respect to the introduced noise level. The maximum error is less than 2.5% with all the stress histories investigated in the current study. It should be noted that the proposed study focuses on the theoretical development of the stress reconstruction methodology. Further investigation is required for the experimental validation studies.

## 4. Conclusions

In this study, an integrated fatigue life evaluation method for structural health monitoring using limited sensor data is developed. Sparse and remote strain gauge measurements from the existing structural health monitoring (SHM) system can be directly used to reconstruct the strain and stress responses for critical spots without direct sensor measurements. The proposed method extends the previous empirical mode decomposition (EMD)-based time-domain reconstruction method by performing real-time fatigue life evaluation for structural health monitoring purposes. The strain/stress reconstruction method based on empirical mode decomposition (REMD) is employed in the proposed method to extrapolate the strain and stress responses using the derived strain and stress transformation function. Mode superposition is used to obtain the strain and stress responses of the critical spot for fatigue life prediction. The overall method is demonstrated using a three-dimensional frame structure and a more complex 19956-DOF simplified airfoil structure. Fatigue analysis using the reconstructed stress responses is performed to demonstrate the effectiveness of the overall method. Based on the current study, several conclusions are drawn:

1. In this study, a novel structural fatigue life evaluation method is proposed to provide prompt, informed fatigue damage predictions of the structures based on limited strain gauge measurement. Using this method, the evolution of fatigue damage within structures can be performed using the limited strain gauge measurement.
2. According to numerical analysis results, the proposed method can produce results which are very close to theoretical solutions considering a practical noisy measurement system. The reconstructed results have an overall correlation coefficient larger than 0.975 under 10% RMS noise settings.
3. Fatigue crack growth analysis using the reconstructed stress responses agrees well with the crack size predictions using the original stress responses. The crack size prediction using the reconstructed stress responses is not sensitive to the introduced noise.

However, limitations should be stated as follows: Firstly, in the current study, locations and damage severity of critical spots are assumed to be known from either previous field data or damage detection results. On the other hand, a crack will definitely alter the reconstructed dynamic responses when it is large enough to affect the structure parameters such as stiffness and natural frequency. From this point of view, the reconstructed responses containing damage information can be used as damage detection. However, damage detection (including locating and evaluating the severity of the damage) is beyond the scope of this paper and is listed as future work. Secondly, if the high damping C is introduced, the amplitude of the measured strain signal will reduce rapidly. Then, the mode response may be extracted incompletely from the measured strain signal which will lead to the uncertainty of the strain reconstruction method.

The current study focuses on theoretical development, and further study is thus required to experimentally validate the proposed methodology. Only mode I planar crack is considered in the demonstrated examples. Curvilinear or more general 3D cracks need further investigations, and the uncertainty of the loading on the fatigue life evaluation is also an interesting study for the future.

## Figures and Tables

**Table 1:** Properties of structural member elements.

Property	Value
Cross-section area B (m^2^)	0.01
Moment of inertia I_y_ (m^4^)	8.3333 × 10^−6^
Moment of inertia I_z_ (m^4^)	8.3333 × 10^−6^
Torsion constant J (m^4^)	6.6667 × 10^−5^
Young’s modulus E (GPa)	200
Poisson’s ratio *ν*	0.3
Shear modulus G (GPa)	E/(2 + 2*ν*)
Mass per unit volume *ρ* (kg/m^3^)	7.8 × 10^−3^

**Table 2:** Properties of FE model in ANSYS.

Property	Value
Material	aluminum 7075
Element type	solid185
Young’s modulus E (GPa)	72
Poisson’s ratio *ν*	0.33
Mass per unit volume *ρ* (kg/m^3^)	2.81 × 103
Number of element	14951
